# Supplementary material for: Differential Gene Expression in Porcine Lung Compartments after Experimental Infection with Mycoplasma hyopneumoniae
Source: Animals (Basel). 2024 Apr 25;14(9):1290. doi: 10.3390/ani14091290 (PMC11083927; doi:10.3390/ani14091290)
Supplement: Supplementary file 1 [file animals-14-01290-s001.zip › animals-2933086-supplementary.pdf]

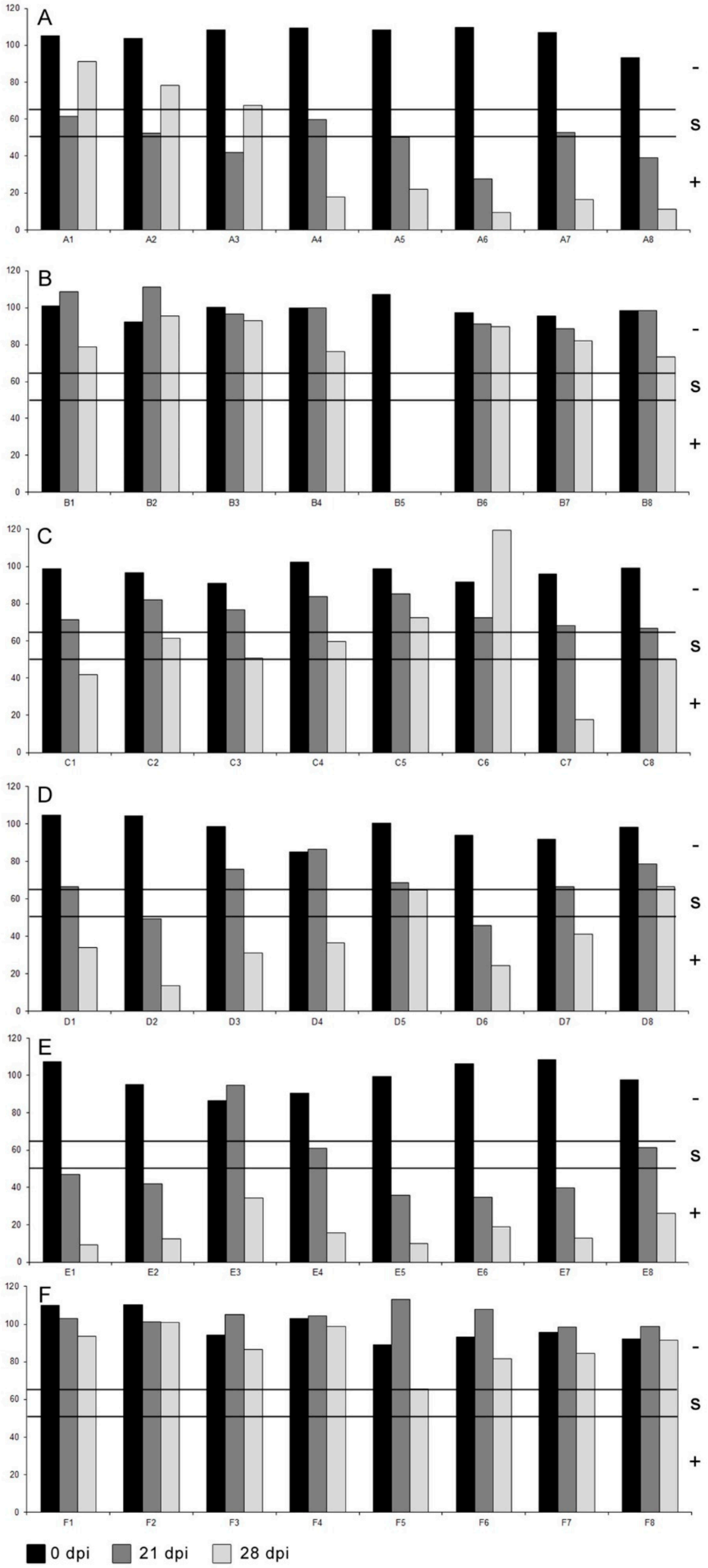
A
B
C
D
E
F
-
S
+
A1 A2 A3 A4 A5 A6 A7 A8
B1 B2 B3 B4 B5 B6 B7 B8
C1 C2 C3 C4 C5 C6 C7 C8
D1 D2 D3 D4 D5 D6 D7 D8
E1 E2 E3 E4 E5 E6 E7 E8
F1 F2 F3 F4 F5 F6 F7 F8
0 dpi
21 dpi
28 dpi

Figure S1: Mhyo blocking ELISA results for animal groups A to F. Y axis represents percentages of optical density (OD) in comparison to the buffer control. -: Negative results (>65%). s: Suspect results (65%-50%.) +: Positive results (<50%). Animal ID B5 had to be euthanized at 10 dpi based on the humane endpoint scoring sheet associated to this animal study. Non-specific clinical signs and moderate respiratory distress were observed prior to the euthanasia of the animal. Post-mortem examination did not reveal specific pathological changes. Mhyo was the sole porcine pathogen isolated from the respiratory tract.
